# PrEP knowledge, attitudes, and perceived barriers to access among American Indian/Alaska Native people in the US: Results from an online survey

**DOI:** 10.1371/journal.pone.0321422

**Published:** 2025-04-30

**Authors:** Sarah T. Roberts, Sarah M. Hatcher, Erica N. Browne, Brigg Reilley, Matthew Bensen, Andrew Freeman, Bob Henne, Ashley Hoover, Monica M. Desjardins, Jessica Leston

**Affiliations:** 1 Women’s Global Health Imperative, RTI International, Oakland, California, United States of America; 2 Applied Public Health Research Center, RTI International, Durham, North Carolina, United States of America; 3 Northwest Portland Area Indian Health Board, Portland, Oregon, United States of America; 4 Center for Survey Research Engineering, RTI International, Durham, North Carolina, United States of America; 5 Center for Communication and Media Impact, RTI International, Durham, North Carolina, United States of America; University of Technology Sydney, AUSTRALIA

## Abstract

**Introduction:**

Compared to non-Indigenous communities*,* American Indian/Alaska Native (AI/AN) people are inequitably impacted by HIV, yet few data are available on barriers to pre-exposure prophylaxis (PrEP) use in this population. This study sought to examine PrEP knowledge, attitudes, and perceived barriers to use among AI/AN people in the United States.

**Methods:**

A cross-sectional, online survey was administered from January-May 2023 to respondents ≥ 16 years of age who identified as AI/AN. The survey assessed sociodemographic characteristics, PrEP knowledge, attitudes towards PrEP, and experiences with and barriers to PrEP use. Sociodemographic correlates of PrEP knowledge and attitudes were identified using bivariable and multivariable regression models.

**Results:**

The survey enrolled 403 participants and 354 (87.8%) completed all questions. Respondents had relatively low PrEP knowledge (mean score 4.0 of 9, standard deviation [SD] 3.0). Few (7%) had ever used PrEP. Mean scores on the stigma scales were 2.1 of 5 for stigmatizing PrEP attitudes (SD 0.7), 2.4 of 5 for anticipated stigma (SD 0.56), and 3.0 of 5 for perceived stigma (SD 0.38). Among non-users, 43.1% were not sure if they would be able to get a PrEP prescription if they desired, and 2.7% believed they would not be able to get one. The most common perceived barriers were not knowing where to get PrEP (54.7%) and concerns around discomfort, judgement, and privacy at the health facility (27.3%). In adjusted models, living on tribal/reservation lands was significantly associated with lower PrEP knowledge, higher stigmatizing attitudes, and higher anticipated stigma, and lower PrEP knowledge was associated with higher stigmatizing attitudes and anticipated stigma. Age, gender identity, sexual orientation, urban residence, and strength of connection to indigenous culture were also significantly correlated with one or more outcomes.

**Conclusions:**

Our findings underscore the need for widespread sensitization about PrEP in Indigenous communities and for strategies to improve PrEP access and reduce stigma from providers and community members.

## Introduction

*There are over 574 federally recognized Tribal Nations in the United States, and even more state recognized Nations and Tribal Nations and communities colonially disposed of recognition. Each of these Tribes have their own culture, history, knowledge and way of referring to themselves. Throughout this article, we will be using the term American Indian/Alaska Native (AI/AN) to refer to the Indigenous people of the United States, including those who identify as Indigenous, American Indian, Alaska Native, Native American, Native Hawaiian, or Pacific Islander.*

American Indian and Alaska Native (AI/AN) people experience historical and ongoing traumas and inequities. Since their arrival, European settlers have worked to destroy Indigenous nations and cultures through land removal, forced relocation, boarding schools, physical and biological warfare, and failures to fulfill treaty obligations [1–5]. Colonization and genocide have produced intergenerational trauma, violence, discrimination, and poverty that, in turn, affect access to quality health care, housing and food security, education, and culturally appropriate support services [6–9] and result in higher disease burden in Indigenous communities compared to non-Indigenous communities, including a disproportionate burden of HIV [10–16]. While HIV diagnostic trends for most other races in the United States (US) remained stable or declined between 2018 and 2022, AI/AN populations saw a 30% increase [17]. The majority of new diagnoses is attributed to male-to-male sexual transmission [18]. In 2022 the rate of new HIV diagnosis among AI/AN people was 10.6 per 100,000, twice the rate of 5.3 per 100,000 for white populations [17]. AI/AN people living with HIV are also least likely to be aware of their status.

Daily oral pre-exposure prophylaxis (PrEP) is a biomedical HIV prevention strategy that has been shown to be up to 99% effective at preventing HIV acquisition during sex and at least 74% effective at preventing HIV from injection drug use [19,20]. Because sample sizes for AI/AN are usually too small to support analyses, there are few data available on awareness, coverage, or barriers to use of HIV pre-exposure prophylaxis (PrEP) in this population, or with a focus on Native Two Spirit, lesbian, gay, bisexual, transgender, queer, or any other gender or sexual minority (2SLGBTQ+) health [21,22]. One 2019 study conducted among sexual minority men found that AI/AN men were significantly less likely than White men to have ever used PrEP [23]. Previous research has shown the risk of HIV acquisition is largely dictated by social and economic factors such as lack of access to healthcare, lower health literacy regarding risk and effective prevention methods, financial constraints, legal protections, and social stigmas that lower self-efficacy and acceptability of prevention methods [24]. In particular, PrEP stigma arises from its perceived associations with drug use, multiple sexual partnership, sexual orientation, or gender identity; or from the misunderstanding that PrEP is HIV treatment rather than prevention [25]. Although AI/AN individuals may have access to PrEP at no out-of-pocket cost, several barriers may still hinder their ability to receive comprehensive PrEP care. These challenges include limited funding to the Indian Health System and high vacancy rates and staff turnover. In addition, there is a need for culturally responsive information and services, a lack of which could restrict meaningful access to PrEP education, counseling, and prescriptions [26].

Previous studies in the US and globally have shown that low knowledge and awareness of PrEP, PrEP stigma, and healthcare costs and accessibility are all common barriers to PrEP uptake and continuation [27–30]. However, PrEP use has not been adequately studied among AI/AN populations, and the relevance and importance of these barriers may differ for Indigenous communities due to the aforementioned historical trauma and disparities in education, healthcare access, and economic security, as well as the unique aspects of Indigenous culture and beliefs. Additional research addressing PrEP use among AI/AN people is needed to inform efforts to support PrEP uptake and adherence. The current study sought to examine PrEP knowledge, attitudes, and perceived barriers to use among AI/AN people living in the US.

## Methods

### Study design, population, and procedures

The study was a cross-sectional, online survey. To be eligible, respondents had to be ≥ 16 years of age, be able to read and respond to the survey in English, and identify as AI/AN, including Indigenous, Native American, Native Hawaiian, or Pacific Islander.

Participants were recruited via a convenience sampling approach, leveraging the reach of email, text message, and Instagram platforms managed by the Northwest Portland Area Indian Health Board (NPAIHB), each of which had between 140 and 6,610 subscribers. These platforms included a mix of nationwide and local audiences, including youth, people identifying as 2SLGBTQ + , health educators, and clinicians. The first wave of recruitment was limited to e-mail and text message distribution lists to help prevent fraudulent activity in the survey. A general message with information about the survey was sent to each email listserv, after which separate personalized messages with unique survey links were sent to each subscriber’s address. Recruitment via short message service (SMS) was conducted by sending a mass text message about the survey asking interested recipients to text a phrase to a specific phone number, and then texting a unique link to those who responded. In a second wave of recruitment through Instagram, posts to the NPAIHB platforms provided information about the survey and asked interested viewers to text a phrase to a specific phone number to receive a unique link. Potential participants could also receive a unique survey link through referral from other invitees or directly requesting one from NPAIHB.

By clicking on their unique survey link, respondents were taken to the survey landing page, which included a brief consent form to screen for the survey. Those who consented were directed to a secure web-based data collection system (Voxco) to begin a self-administered screener. Respondents were notified if they qualified for the survey once the screener was completed to avoid revealing eligibility criteria. Eligible respondents were then encouraged to participate by mention of an Amazon gift card and directed to an online consent form. If consent was granted, they proceeded to the full survey.

### Data management, fraud detection, and security

Google’s reCAPTCHA V3 was used to help screen out robot survey completions (‘bots’) from the survey. All screener and survey data files were monitored daily by a staff member to flag potentially fraudulent activity based on survey response patterns and respondent metadata. Such activity could include multiple entries from the same IP address or the same or very similar email addresses, mismatched age and date of birth, and invalid responses to open-ended questions on Tribe name, favorite Native foods, and the first Native medicine they were introduced to. The study team met weekly to review flagged responses and make a final determination on validity. Screener and survey data stored separately on secure servers at the RTI data management center with access controlled via role-based security. A deidentified dataset was created for analysis and stored on a separate network, also with limited access.

### Measures

The screener and main surveys are included in Supplemental Appendix 1 in S1 File. Sociodemographic characteristics included age in years, gender identity, sexual orientation, geographic residence (state, urbanicity, and whether on reservation/tribal lands), type of healthcare facility most often utilized (private, Indian Health Service (IHS), Tribal, or Urban Indian facility), and self-reported strength of connection to Indigenous culture or identity.

PrEP knowledge was assessed with 9 of 13 questions developed by Walsh et al. [31]. Response options were true, false, or don’t know. Correct responses were coded as 1 and incorrect and ‘don’t know’ responses coded as a 0. A summary score was created based on the number of correct responses. After these questions were completed, participants were asked to watch a video about PrEP, developed by the CDC (https://youtu.be/1_eo17YahCo) or read its transcript before responding to the remaining questions. Respondents could not return to the knowledge questions after watching the YouTube video or reading its transcript.

PrEP attitudes were measured with a set of four scales. Response options for all items used a 5-point Likert scale ranging from strongly disagree (1) to strongly agree (5). Items with opposite valence were reverse coded, then scores for each scale were calculated as the mean of responses.

- Positive PrEP attitudes (alpha = 0.61) were measured with the 3-item subscale of the PrEP Stigma and Positive Attitudes scale [32]. This scale was dropped from subsequent analyses due to the relatively low internal consistency and lack of correlation with the stigmatizing PrEP attitudes scale (rho = -0.03), which should have had a strong, negative correlation.
- Stigmatizing PrEP attitudes are the negative attitudes or beliefs that the respondent has about PrEP use. These were measured with the 7-item subscale from the PrEP Stigma and Positive Attitudes scale (alpha = 0.83) [32].
- Anticipated PrEP stigma is defined as expectations of being treated negatively because of their PrEP use and was measured with 9 items adapted from the HIV Pre-exposure Prophylaxis Stigma Scale (alpha = 0.77) [33].
- Perceived PrEP stigma is defined as the negative attitudes a person believes that other people hold about PrEP users and was measured with a scale developed by the authors, because we were not aware of any existing validated scales. We adapted the questions from the three scales described above to ask whether the respondent thought people in their community would agree with the statement, e.g., “*Most people in my community think that people on PrEP are irresponsible*”, “*Most people in my community would assume I was HIV positive if I took PrEP*” (19 items, alpha = 0.90).

To assess PrEP experiences and barriers, respondents were asked if they were currently, or had ever taken PrEP for HIV prevention. If currently taking PrEP, they were asked how easy or hard they found it take PrEP on a 5-point Likert scale. If they had never taken it, they were asked about their interest in using PrEP, measured on a 5-point Likert scale; whether they thought they would be able to get a prescription if they wanted one (yes/no/not sure); and, if they responded no or were unsure, why they thought they would not be able to get a prescription (selecting all that apply from a pre-populated list with an ‘other, specify’ option).

### Data analyses

The primary analyses included all 354 participants who completed the survey. An additional 23 enrolled participants (5.9%) completed the first set of questions, on PrEP knowledge, and subsequently discontinued the survey. These were included in analyses of the PrEP knowledge outcome to maximize representativeness. Descriptive statistics were calculated for demographic characteristics of survey respondents, knowledge and stigma scores, and barriers to PrEP use. A negative binomial model was used to compare the number of correct PrEP knowledge questions by participant characteristics. Linear regression models were used to estimate differences in mean stigma scores by participant characteristics. Measures that were found to be associated with stigma scores at p < 0.05 were adjusted for other demographics in multivariable models, including age (categorized into tertiles), sexual orientation, gender identity, and living on reservation or tribal lands. PrEP knowledge was also examined as a correlate of personal attitudes towards PrEP (i.e., stigmatizing attitudes and anticipated stigma). All analyses were conducted using Stata version 17.

### Ethics Statement

The study protocol was reviewed and approved by the Portland Area Indian Health Service Institutional Review Board. All participants provided informed consent in English by reading the full consent form on the survey website and checking a box indicating that they agree to participate in the study. A waiver of parental consent was granted for minors aged 16–17 years. Respondents who completed the survey received a $100 e-gift card in appreciation for their time and for sharing their knowledge. The amount of the gift card was not disclosed until after completion of the survey.

## Results

### Participant characteristics

From January 23 to April 28 2023, 711 people accessed the survey and consented to screening, 489 (68.8%) completed the screener and were eligible for the study, and 403 (82.4%) eligible participants consented to the main survey. Among those who consented, 10 were determined to be fraudulent and excluded from the survey, 377 (93.5%) completed at least the first set of questions about PrEP knowledge, and 354 (87.8%) completed the entire survey. Median age among the participants who completed the survey was 41 years (interquartile range [IQR] 30–50; Table 1). The majority identified as women only (60.5%) and straight or opposite-gender loving (62.1%), but participants reported a wide range of gender identities and sexual orientations. About half of participants lived in the Western US, one third lived on tribal or reservation lands (30.8%), and half (50.6%) lived in urban areas.

**Table 1:** Demographic characteristics of participants.

Characteristic	Survey completion			
	All	PrEP knowledge questions only	Total	p-value
	N (%)	N (%)	N (%)	
**Total**	354 (100)	23 (100)	377 (100)	
**Age - *median (IQR)***	41 (30-50)	48 (39-58)	41 (31-50)	0.01
**Race/ethnicity**				0.08
Indigenous alone	229 (64.7)	19 (82.6)	248 (65.8)	
Indigenous in combination	125 (35.3)	4 (17.4)	129 (34.2)	
**Gender identity**				0.05
Woman	214 (60.5)	14 (60.9)	228 (60.5)	
Man	65 (18.4)	8 (34.8)	73 (19.4)	
Gender diverse^*^	75 (21.2)	1 (4.3)	76 (20.2)	
**Sexual orientation**				0.26
Straight (opposite-gender loving)	220 (62.1)	17 (73.9)	237 (62.9)	
Non-straight^**^	134 (37.9)	6 (26.1)	140 (37.1)	
**Geographic region**^				0.26
Alaska	20 (5.7)	3 (13)	23 (6.1)	
East	23 (6.5)	1 (4.3)	24 (6.4)	
Northern Plains West	20 (5.7)	2 (8.7)	22 (5.9)	
Northern Plains East	24 (6.8)	3 (13)	27 (7.2)	
Southern Plains	30 (8.5)	0 (0)	30 (8)	
Southwest	59 (16.7)	6 (26.1)	65 (17.3)	
West	177 (50.1)	8 (34.8)	185 (49.2)	
**Geographic area**				0.51
Urban	179 (50.6)	10 (43.5)	189 (50.1)	
Rural or suburban	175 (49.4)	13 (56.5)	188 (49.9)	
**Living on Tribal lands/reservation**				0.97
Yes	109 (30.8)	7 (30.4)	116 (30.8)	
No	245 (69.2)	16 (69.6)	261 (69.2)	
**Healthcare facility type**				0.27
Private facility	133 (37.6)	6 (26.1)	139 (36.9)	
Federal (IHS), Tribal, or Urban Indian	221 (62.4)	17 (73.9)	238 (63.1)	
**Connection to Indigenous culture/identity**				0.48
Not Strong at all	10 (2.8)	1 (4.4)	11 (2.9)	
Somewhat Strong	77 (21.8)	5 (21.7)	82 (21.8)	
Neutral	32 (9.0)	1 (4.4)	33 (8.8)	
Strong	137 (38.7)	6 (26.1)	143 (37.9)	
Very Strong	98 (27.7)	10 (43.5)	108 (28.7)	
**PrEP Use**				
Ever used PrEP	25 (7.1)	1 (4.4)	26 (6.9)	0.74
Currently using PrEP	11 (3.1)	1 (4.4)	12 (3.2)	
**PrEP knowledge score** – ***median (IQR)***	4 (1-6)	2.6, 2 (0-5)	4.0, 4 (1-6)	0.02

*Diverse gender identities included gender fluid (n = 9), genderqueer (n = 7), Indigiqueer (n = 24), non-binary (n = 26), other (n = 5), questioning or unsure (n = 7), transgender (n = 6) transman (n = 4), transwoman (n = 3), and Two Spirit (n = 47). Respondents could choose multiple identities.

** Non-straight sexual orientations included asexual (n = 3), bisexual (n = 57), gay (n = 24), Indigiqueer (n = 22), lesbian (n = 7), other (n = 5), pansexual (n = 18), queer (n = 35), and questioning (n = 8). Respondents could choose multiple sexual orientations.

^ *East*: Alabama, Connecticut, Florida, Georgia, Illinois, Indiana, Louisiana, Maine, Maryland, Massachusetts, Mississippi, Missouri, New York, North Carolina, Pennsylvania, Ohio, Rhode Island, South Carolina, Tennessee, Virginia; *Northern Plains West*: Montana, Nebraska, North Dakota, South Dakota, Wyoming; *Northern Plains East:* Iowa, Michigan, Minnesota, and Wisconsin; *Southern Plains*: Kansas, Oklahoma, and Texas; *Southwest:* Arizona, Colorado, Nevada, New Mexico, Utah; *West*: California, Hawaii, Idaho, Oregon, Washington [34].

Participants who discontinued after completing the knowledge questions had significantly lower PrEP knowledge scores, were significantly older, and were somewhat less likely to identify as gender-diverse (Table 1).

### PrEP practices

Only 7.1% of respondents (n = 25) had ever used PrEP and 3.1% were currently using it (n = 11), of whom 81.8% (n = 9) found taking PrEP easy or very easy. Among those who had never used PrEP (n = 329), 64.4% felt it wasn’t a good option for them and 12.2% were unsure, while 17.3% wanted to think more about it and 5.8% were ready to get screened or initiate (Table 1).

### PrEP knowledge

Overall, respondents had low levels of PrEP knowledge, with a mean of 4 out of 9 questions answered correctly. Only 6% (n = 22) answered all questions correctly, and 23% (n = 86) answered none of the questions correctly. The distribution of responses to the individual items is shown in S1 Fig. The most common response for most questions was “Don’t Know” (35.0%-59.8%). Three questions were answered correctly by a majority of participants, on what PrEP is (59.7%), its effectiveness (55.1%), and whether it protects against STIs (58.9%). The questions most often answered incorrectly pertained to use of PrEP as HIV treatment (26.1%), severity of side effects (19.7%), and using PrEP without knowing your status (17.0%).

In both bivariate analysis and after adjusting for age, gender identity, sexual orientation, and residence on tribal lands (Table 2), sociodemographic correlates of higher PrEP knowledge scores were identifying as non-straight vs. straight (adjusted ratio [aR] 1.42; 95% confidence interval [CI] 1.19, 1.68; p < 0.001) and currently (aR 1.50; 95% CI 1.27, 1.78; p < 0.001) or formerly using PrEP (aR 1.78, 95% CI 1.46–2.16, p < 0.001), versus never having used PrEP. Lower knowledge scores were correlated with older age (aR for oldest tertile vs. youngest: 0.75; 95% CI 0.61, 0.92; p = 0.005), living in rural or suburban vs. urban areas (aR 0.79, 95% CI 0.66–0.93, p = 0.005), and living on tribal or reservation lands vs. not (aR 0.83; 95% CI 0.70, 0.99; p = 0.04).

**Table 2:** Sociodemographic correlates of PrEP knowledge (n = 377; score range 0-9).

Characteristic	Score Mean (SD); Median (IQR)	Unadjusted associations R (95% CI), p-value	Adjusted associations^*^ aR (95% CI), p-value
Overall sample	4.0 (3.0); 4 (1-6)		
Age			
Age tertile 1 (16–34)	4.9 (2.8); 5 (3-7)	ref	ref
Age tertile 2 (35–46)	3.9 (3.0); 4 (0.5-6)	**0.80 (0.68, 0.94), 0.008**	0.89 (0.75, 1.06), 0.20
Age tertile 3 (47–75)	3.1 (3.9); 3 (0-6)	**0.64 (0.53, 0.78), < 0.001**	**0.75 (0.61, 0.92), 0.005**
Gender identity			
Woman only	3.7 (2.9); 4 (1-6)	Ref	Ref
Man only	4.5 (3.1); 5 (1-7)	**1.21 (1.02, 1.44), 0.03**	1.05 (0.85, 1.30), 0.65
Gender diverse	4.7 (2.8); 5 (2-7)	**1.26 (1.01, 1.57), 0.04**	1.05 (0.86, 1.28), 0.66
Sexual orientation			
Identify as straight	3.3 (2.8); 3 (0-6)	Ref	Ref
Non-straight	5.3 (2.8); 6 (3-8)	**1.59 (1.39, 1.83), < 0.001**	**1.42 (1.19, 1.68), < 0.001**
Geographic area			
Urban	4.7 (2.9); 5 (2-7)	Ref	
Rural or suburban	3.3 (2.8); 3 (0-6)	**0.70 (0.60, 0.82), < 0.001**	**0.79 (0.66, 0.93), 0.005**
Living on Reservation/ Tribal Lands			
Yes	3.3 (2.9); 3 (0-6)	**0.78 (0.65, 0.93), 0.006**	**0.83 (0.70, 0.99), 0.04**
No	4.3 (2.9); 5 (2-6)	Ref	Ref
Healthcare facility type			
Private	4.2 (3.0); 5 (1-7)	1.11 (0.95, 1.29), 0.18	–
Federal (IHS), Tribal, or Urban Indian	3.8 (3.0); 4 (1-6)	Ref	
Connection to Indigenous identity or culture			
Very strong	3.8 (2.8); 4 (1-6)	0.99 (0.38, 0.49), 0.91	–
Strong	4.2 (3.1); 5 (1-7)	1.09 (0.91, 1.30), 0.36	
Neutral, somewhat strong, or not at all strong	3.9 (2.9); 4 (1-6)	Ref	
PrEP use status			
Current	7.3 (1.5); 7.5 (6-8.5)	**1.91 (1.66, 2.20), < 0.001**	**1.50 (1.27, 1.78), < 0.001**
Past	7.1 (1.8); 8 (7-8)	**1.88 (1.61, 2.20), < 0.001**	**1.78 (1.46, 2.16), < 0.001**
Never but interested	3.5 (2.5); 3 (2-6)	0.93 (0.68, 1.28), 0.65	0.93 (0.66, 1.31), 0.67
Never but not interested or unsure	3.8 (2.9); 4 (1-6)	Ref	Ref

*Adjusted for age, gender identity, sexual orientation, and living on tribal lands

Bold text indicates p < 0.05. aR: adjusted ratio; CI: confidence interval; IQR: interquartile range; R: ratio; SD: standard deviation

### Respondents’ PrEP attitudes

Mean scores on the Stigmatizing PrEP Attitudes scale were low (mean 2.1 of 5, SD 0.7), corresponding to disagreement with most stigmatizing statements. In fact, over half of participants disagreed or strongly disagreed with each individual item. The item with the highest level of agreement was “Sex with someone on PrEP is risky”, to which 13.5% agreed or strongly agreed (S2 Fig).

In bivariate models, mean scores on the Stigmatizing PrEP Attitudes scale were significantly correlated with most sociodemographic factors we examined except for age, PrEP use, and connection to indigenous culture (Table 3). However, most associations became nonsignificant after adjustment for age, gender identity, sexual orientation, and residence on tribal or reservation lands. Stigmatizing attitudes remained significantly higher among respondents identifying only as men vs. only as women (adjusted beta [aβ] 0.32; 95% CI 0.12, 0.51; p = 0.001) and among those living on tribal or reservation lands vs. not (aβ 0.20; 95% CI 0.05, 0.35; p = 0.01). Additionally, higher PrEP knowledge scores were significantly associated with lower Stigmatizing PrEP Attitudes scores (aβ -0.06; 95% CI -0.08, -0.03; p < 0.001).

**Table 3:** Sociodemographic correlates of Stigmatizing PrEP Attitudes (n = 377; score range 1-5).

Characteristic	Score Mean (SD); Median (IQR)	Unadjusted associations β (95% CI), p-value	Adjusted associations^*^ aβ (95% CI), p-value
Overall sample	2.1 (0.7); 2.1 (1.7-2.6)		
Age			
Age tertile 1 (16–34)	2.0 (0.8); 2.0 (1.4-2.4)	ref	
Age tertile 2 (35–46)	2.1 (0.6); 2.1 (1.7-2.6)	0.06 (-0.11, 0.22), 0.52	0.06 (-0.11, 0.24), 0.49
Age tertile 3 (47–75)	2.2 (0.6); 2.1 (1.7-2.6)	0.17 (0.00, 0.35), 0.05	0.14 (-0.04, 0.32), 0.14
Gender identity			
Woman	2.1 (0.6); 2.1 (1.7-2.6)	Ref	Ref
Man	2.4 (0.8); 2.3 (1.9-2.9)	**0.26 (0.07, 0.45), 0.006**	**0.32 (0.12, 0.51), 0.001**
Gender diverse	1.9 (0.6); 1.9 (1.4-2.3)	**-0.25 (-0.43, 0.08), 0.004**	-0.08 (-0.31, 0.15), 0.48
Sexual orientation			
Identify as straight	2.0 (0.7); 1.9 (1.4-2.3)	Ref	Ref
Non-straight	2.2 (0.7); 2.1 (1.7-2.6)	**0.24 (0.09, 0.39), 0.001**	-0.13 (-0.31, 0.06), 0.18
Geographic area			
Urban	2.0 (0.7); 2.0 (1.6-2.4)	Ref	Ref
Rural or Suburban	2.3 (0.7); 2.3 (1.9-2.7)	**0.14 (0.005, 0.29), 0.04**	0.05 (-0.12, 0.21), 0.56
Living on Reservation/ Tribal Lands			
Yes	2.3 (0.7); 2.3 (1.9-2.7)	**0.25 (0.09, 0.40), 0.002**	**0.20 (0.05, 0.35), 0.01**
No	2.0 (0.7); 2.0 (1.6-2.4)	**Ref**	**Ref**
Healthcare facility type			
Private facility	2.0 (0.6); 2.0 (1.6-2.4)	**-0.16 (-0.31, -0.02), 0.03**	-0.10 (-0.25, 0.05), 0.21
Federal (IHS), Tribal, or Urban Indian	2.2 (0.7); 2.1 (1.7-2.6)	Ref	
Connection to indigenous culture			–
Very strong	2.1 (0.7); 2.1 (1.7-2.6)	-0.17 (-0.34, 0.00), 0.05	
Strong	2.0 (0.6); 2.0 (1.6-2.4)	-0.09 (-0.27, 0.09), 0.33	
Neutral, somewhat strong, or not at all strong	2.2 (0.7); 2.1 (1.7-2.6)	ref	
PrEP use status			–
Current	2.1 (1.3); 1.6 (1.2-2.5)	0.06 (-0.35, 0.47), 0.78	
Past	2.0 (0.7); 1.8 (1.6-2.1)	-0.12 (-0.50, 0.25), 0.52	
Never but interested	2.2 (0.7); 2.0 (1.7-2.7)	0.06 (-0.26, 0.38), 0.70	
Never but not interested or unsure	2.1 (0.7); 2.1 (1.7-2.6)	Ref	
PrEP knowledge score	N/A	**-0.07 (-0.09, -0.05), < 0.001**	**-0.06 (-0.08, -0.03), < 0.001**

*Adjusted for age, gender identity, sexual orientation, and living on tribal lands

Bold text indicates p < 0.05. aβ: Adjusted beta; CI: confidence interval; IHS: Indian Health Service; IQR: interquartile range; SD: standard deviation

### Perceived and anticipated stigma.

Participants perceived low levels of PrEP knowledge among their community members: 72.6% disagreed or strongly disagreed that most people in their community know what PrEP is. Mean scores on the Anticipated and Perceived PrEP Stigma scales corresponded to neutral or non-stigmatizing responses on most items: 2.4 of 5 (SD 0.6) on the Anticipated Stigma Scale and 3.0 of 5 (SD 0.5) on the Perceived Stigma Scale. For anticipated stigma, the only item to which >20% agreed or strongly agreed was that people may experience problems when they tell their sex partner(s) they are taking PrEP (43.4%). (S3 Fig). On the Perceived PrEP Stigma scale, the individual items with the highest rates of agreement pertained to associations of PrEP use with being HIV-positive (50.4%) or LGBTQ2S+ (57.1%), using drugs (37.6%), or sleeping with many people (36.2%). (S4 Fig).

In adjusted models, anticipated PrEP stigma scores were significantly lower among respondents who did not identify as straight (aβ -0.20; 95% CI -0.36, -0.05; p = 0.01) and who reported a strong connection to indigenous culture vs. a neutral, somewhat strong, or not at all strong connection (aβ -0.18; 95% CI -0.33, -0.04; p = 0.02; Table 4). Participants reporting a very strong connection had score differences of a comparable magnitude that were not significant (aβ -0.13; 95% CI -0.26, 0.01; p = 0.06). PrEP knowledge scores were significantly, negatively correlated with anticipated PrEP stigma (aβ -0.04; 95% CI -0.06, -0.02; p < 0.001). Perceived PrEP stigma was significantly higher among respondents living on tribal or reservation lands (aβ 0.12; 95% CI 0.02, 0.23; p = 0.03), and significantly lower among men compared to women (aβ -0.21; 95% CI -0.35, -0.07; p = 0.003) and among respondents who had formerly used PrEP (aβ -0.28, 95% CI -0.55 to -0.01, p = 0.04) or had never used it but were interested (aβ -0.28; 95% CI -0.50, -0.06; p = 0.01), compared to those who had never used and were not interested or were unsure (Table 5). However, there was no association between current PrEP use and perceived stigma scores.

**Table 4:** Sociodemographic correlates of anticipated PrEP stigma (n = 377; score range 1-5).

Characteristic	Score Mean (SD); Median (IQR)	Unadjusted associations β (95% CI), p-value	Adjusted associations^*^ aβ (95% CI), p-value
Overall sample	2.4 (0.56); 2.4 (2.0-2.9)		
Age			–
Age tertile 1 (16–34)	2.4 (.60); 2.3 (2.0-2.8)	ref	
Age tertile 2 (35–46)	2.4 (.50); 2.4 (2.1-2.9)	-0.005 (-0.15, 0.14), 0.94	
Age tertile 3 (47–75)	2.5 (.58); 2.6 (2.0-2.9)	0.03 (-0.11, 0.17), 0.68	
Gender identity			
Woman	2.5 (.50); 2.4 (2.1-2.9)	Ref	
Man	2.5 (.68); 2.4 (2.1-2.9)	0.03 (-0.12, 0.18), 0.69	0.03 (-0.13, 0.18), 0.75
Gender diverse	2.2 (.56); 2.1 (1.8-2.6)	**-0.30 (-0.45, -0.16), < 0.001**	-0.16 (-0.35, 0.02), 0.09
Sexual orientation			
Identify as straight	2.5 (.53); 2.6 (2.2-2.9)		
Non-straight	2.3 (.57); 2.2 (1.9-2.6)	**-0.28 (-0.40, -0.16), < 0.001**	**-0.20 (-0.36, -0.05), 0.01**
Geographic area			
Urban	2.4 (0.6); 2.4 (2.0-2.8)	Ref	–
Rural or Suburban	2.5 (0.5); 2.4 (2.1-2.9)	0.10 (-0.02, 0.22), 0.09	
Living on Reservation/ Tribal Lands			
Yes	2.5 (0.5); 2.6 (2.2-2.9)	**0.15 (0.02, 0.27), 0.02**	0.11 (-0.01, 0.23), 0.08
No	2.4 (0.6); 2.4 (2.0-2.8)	Ref	
Healthcare facility type			
Private facility	2.4 (.54); 2.4 (2.1-2.9)	**-0.15 (-0.27, -0.03), 0.02**	-0.10 (-0.23, 0.02), 0.11
Federal (IHS), Tribal, or Urban Indian	2.5 (.58); 2.4 (1.9-2.8)	Ref	
Connection to indigenous culture			–
Very strong	2.4 (.60); 2.4 (2.1-2.9)	**-0.14 (-0.28, -0.01), 0.04**	-0.13 (-0.26, 0.01), 0.06
Strong	2.4 (.54); 2.4 (2.0-2.9)	-0.15 (-0.30, -0.001), 0.05	**-0.18 (-0.33, -0.04), 0.02**
Neutral, somewhat strong, or not at all strong	2.5 (.53); 2.3 (2.0-2.8)	Ref	
PrEP use status			–
Current	2.2 (.65); 2.0 (1.4-2.8)	-0.28 (-0.61, -0.06), 0.11	
Past	2.2 (.51); 2.3 (1.9-2.4)	-0.22 (-0.52, 0.08), 0.15	
Never but interested	2.3 (.60); 2.1 (2.0-2.9)	-0.16 (-0.42, 0.09), 0.21	
Never but not interested or unsure	2.5 (.55); 2.4 (2.1-2.9)	Ref	
PrEP knowledge score	N/A	**-0.05 (-0.07, -0.03), < 0.001**	**-0.04 (-0.06, -0.02), < 0.001**

*Adjusted for age, gender identity, sexual orientation, and living on tribal lands

Bold text indicates p < 0.05. aβ: adjusted beta; CI: confidence interval; IHS: Indian Health Service; IQR: interquartile range; SD: standard deviation

**Table 5:** Sociodemographic correlates of perceived PrEP stigma (n = 377; score range 1-5).

Characteristic	Score Mean (SD); Median (IQR)	Unadjusted associations β (95% CI), p-value	Adjusted associations^*^ aβ (95% CI), p-value
Overall sample	3.0 (0.38); 3.0 (2.7-3.2)		
Age			
Age tertile 1 (16–34)	3.0 (.57); 3.1 (2.7-3.4)	ref	–
Age tertile 2 (35–46)	2.9 (.46); 2.9 (2.7-3.2)	-0.09 (-0.21, 0.03), 0.15	
Age tertile 3 (47–75)	2.9 (.38); 3.0 (2.7-3.1)	-0.09 (-0.21, 0.04), 0.16	
Gender identity			
Woman	3.0 (.40); 3.0 (2.8-3.2)	Ref	ref
Man	2.9 (.62); 2.9 (2.4-3.2)	**-0.15 (-0.29, -0.02), 0.02**	**-0.21 (-0.35, -0.07), 0.003**
Gender diverse	3.0 (.54); 2.9 (2.6-3.3)	-0.04 (-0.16, 0.09), 0.55	-0.08 (-0.25, 0.08), 0.29
Sexual orientation			–
Identify as straight	3.0 (.56); 3.0 (2.6-3.3)	Ref	
Non-straight	3.0 (.43); 3.0 (2.7-3.2)	0.01 (-0.10, 0.11), 0.86	
Geographic area			
Urban	2.9 (.53); 2.9 (2.5-3.2)	Ref	–
Rural or Suburban	3.0 (.41); 3.0 (2.9-3.2)	0.11 (0.00, 0.22), 0.05	
Living on Reservation/ Tribal Lands			
Yes	3.1 (.44); 3.0 (2.9-3.2)	**0.11 (0.01, 0.21), 0.03**	**0.12 (0.02, 0.23), 0.03**
No	2.9 (.49); 2.9 (2.6-3.2)	Ref	Ref
Healthcare facility type			
Private facility	2.9 (.49); 2.9 (2.5-3.2)	**-0.11 (-0.21, -0.01), 0.03**	-0.08 (-0.19, 0.03), 0.16
Federal (IHS), Tribal, or Urban Indian	3.0 (.47); 3.0 (2.8-3.3)	Ref	Ref
Connection to indigenous culture			
Very strong	2.9 (.47); 3.0 (2.7-3.2)	-0.04 (-0.15, 0.08), 0.56	–
Strong	3.1 (.49); 2.9 (2.6-3.2)	0.11 (-0.02, 0.24), 0.09	
Neutral, somewhat strong, or not at all strong	3.0 (.49); 3.0 (2.8-3.3)	Ref	
PrEP use status			
Current	2.9 (.65); 3.1 (2.2-3.3)	-0.15 (-0.44, 0.14), 0.31	-0.17 (-0.46, 0.12), 0.25
Past	2.7 (.50); 2.8 (2.6-3.1)	**-0.30 (-0.55, -0.04), 0.02**	**-0.28 (-0.55, -0.01), 0.04**
Never but interested	2.7 (.47); 2.8 (2.6-3.1)	**-0.28 (-0.50, -0.06), 0.01**	**-0.28 (-0.50, -0.06), 0.01**
Never but not interested or unsure	3.0 (.47); 3.0 (2.7-3.2)	Ref	ref

*Adjusted for age, gender identity, sexual orientation, and living on tribal lands

Bold text indicates p < 0.05. aβ: adjusted beta; CI: confidence interval; IHS: Indian Health Service; IQR: interquartile range; SD: standard deviation

### Perceived barriers to PrEP use.

About half of never-users (54.2%) thought they would be able to get a PrEP prescription if they desired, while 43.1% were unsure and 2.7% did not think they would be able to get it. The most common perceived barriers were not knowing where to get PrEP or if it was available at their clinic (54.7%); concerns around comfort talking to providers about PrEP, judgment from providers about PrEP, and lack of privacy at the clinic causing other people to find out they were on PrEP (27.3%); concerns around getting to the clinic such as transport, childcare and opening hours (10.0%); and affordability (10.0%). Respondents who received care at federal (IHS), Tribal, or Urban Indian facilities were more likely to perceive barriers to PrEP prescriptions than those receiving care at private facilities (48.4% vs. 32.3% respectively). They were more likely to report barriers around knowing where to access PrEP (57.9% vs. 46.5%) and around comfort, judgment, and privacy (29.0% vs. 23.3%), but less likely to report barriers around getting to the clinic (8.4% vs. 14.0%) and affordability (9.3% vs. 11.6%).

## Discussion

This cross-sectional online survey assessed PrEP knowledge, attitudes, and perceived barriers to use among AI/AN people across the United States. We found that among respondents who had not used PrEP, only half thought they would be able to get a prescription if they wanted one. The largest barrier to access was not knowing where PrEP was available, followed by concerns about stigma at the health facility, clinic access, and affordability. Respondents had low levels of PrEP knowledge, but their attitudes towards PrEP were mostly neutral or positive, with low to moderate levels of anticipated and perceived stigma. To our knowledge, this is the first study to describe perceptions of PrEP among AI/AN people in the U.S. specifically, and the results can inform efforts to expand access to PrEP and support PrEP use to reduce HIV incidence in this population.

On average, survey respondents answered less than half of the 9 PrEP knowledge questions correctly, almost a quarter answered all questions incorrectly, and “don’t know” was the modal response to most items. Although we did not ask about PrEP awareness directly, PrEP knowledge question responses suggest that most participants were not familiar with PrEP. Additionally, many did not know where to access it, especially if they received care at federal IHS, Tribal, or Urban Indian healthcare facilities. Though we are not aware of data on PrEP knowledge and awareness in the general US population, our findings are comparable to those from studies of population groups who may benefit from PrEP, including heterosexual men and women at high risk of HIV (40% aware of PrEP) [35], people who inject drugs (25% aware of PrEP) [36], men who have sex with men (45%-66% of PrEP knowledge questions answered correctly) [31,37]. Our findings reinforce those from other studies to suggest that additional efforts are needed to increase awareness and accurate knowledge about PrEP.

Stigma and clinic accessibility were also perceived as barriers to getting a PrEP prescription. These issues have been identified in other US populations such as Black women and Black and Latino MSM [38–43]. Studies conducted among providers also confirm that many hold stigmatizing attitudes towards potential PrEP users, due to lack of knowledge about PrEP; assumptions and biases around users’ ability to adhere, sexual behaviors, and risk compensation -- often associated with their race, gender, sexuality, or behaviors that confer HIV risk; and discomfort discussing sexual behavior with clients [44–47]. Concerns around stigma were exacerbated for respondents attending federal (IHS), Tribal, and Urban Indian healthcare facilities, where high provider vacancy rates limit service availability, reduce the time that providers can spend with patients, and lead to contracting of temporary providers, factors which limit providers’ opportunity and motivation to develop trusting relationships with patients that facilitate discussion of sensitive topics [48].

On our scaled measures, most respondents had positive to neutral attitudes about PrEP, and responses to items on perceived and anticipated stigma were largely mixed, with neutral being the most frequent response. The large number of neutral responses may be attributable to low levels of PrEP knowledge; as in other studies, we found that higher knowledge of PrEP was associated with lower scores on measures of stigmatizing PrEP attitudes and anticipated stigma [31,32]. However, the mean scores among our respondents were similar (within 1 SD) to those in studies using the same or similar scales among MSM, transgender women, and people using PrEP [31–33,49]. These populations tend to be more knowledgeable about PrEP, as described above, and to have lower anticipated stigma towards PrEP and more positive PrEP attitudes, most likely stemming from more exposure to PrEP awareness campaigns and to other people using PrEP. Therefore, we might have expected higher levels of stigma among our study respondents because they had less exposure to PrEP. One reason for the generally positive or accepting attitudes in our study may be the strong emphasis on health and wellness in many tribal cultures. A third to half of respondents said they would be perceived to be living with HIV, LGBTQ2S + , using drugs, or having multiple partners if they used PrEP, but very few reported that their family (14%) or friends (4%) would not approve of their PrEP use. In contrast, in a study of PrEP-naïve women in urban Connecticut, similar proportions endorsed PrEP stereotypes around promiscuity and HIV, and a 25%-33% expected disapproval from family and friends [50]. In our study, anticipated stigma scores were also significantly lower among the large proportion of respondents (62%) who reported a strong or very strong connection to indigenous culture, strengthening the hypothesis that stronger engagement in a community that emphasizes health and wellness may reduce PrEP stigma. A study conducted among AI/AN men living with HIV also found that cultural pride and engagement in Indigenous services, religion, and spirituality built resilience to stigma and facilitated ART adherence [51].

By far the most frequently endorsed anticipated stigma item on our survey concerned anticipation of problems with sex partners upon PrEP disclosure: over 40% agreed or strongly agreed, compared to less than 20% for the other items. Multiple studies conducted across a wide range of populations and geographic regions have reported similar concerns around disclosing use of PrEP and other HIV prevention methods to sex partners [38,39,52–54]. Participants in those studies report that their partners will associate their PrEP use with infidelity, promiscuity, or being HIV-positive (misunderstanding that PrEP is actually treatment instead of prevention), or that partners will believe they are being accused of the same behaviors themselves. These fears reveal the underlying truth that PrEP use is stigmatized because of its association with socially unacceptable sexual behaviors and desires [25] and highlight the importance of shifting the narrative to emphasize PrEP use as a strategy to improve sexual health, protect both partners in the relationship, and reduce anxiety about HIV acquisition [55–58].

Two key limitations should be considered when interpreting the study findings. First, because we used a convenience sampling approach, our study population is not representative of all AI/AN people across the United States. Women and older adults were overrepresented, with fewer men and young adults responding. However, our approach allowed us to reach a large number of AI/AN people in a short period of time, was diverse with respect to geography, sexual orientation, and gender identity. Second, we were not able to validate our PrEP attitudes and stigma scales with AI/AN respondents before including them in the survey. As a result, our measure of positive PrEP attitudes had poor psychometric properties and was not considered further for analysis. The other measures appeared to perform well, but there is a risk that some items lacked relevance or were misunderstood. It is also important to note that the current study sought to understand PrEP knowledge, attitudes, and percieved barriers in the AI/AN population at-large, in order to understand community perspectives, and was not restricted to those potentially eligible for PrEP. Future studies should also seek to understand these factors specifically among AI/AN people who could benefit from PrEP use to better tailor interventions to increase uptake and continuation.

Findings from this study have several implications for public health actions to improve PrEP access and use among AI/AN communities in the US. Efforts to raise awareness and increase knowledge about PrEP should be intensified and expanded to reach the broader community (in addition to people who would benefit from PrEP directly) to reduce stigmatizing attitudes and build sources of social support for PrEP users.[38,59–62] Sensitization efforts should especially aim to engage older adults, those in living in nonurban areas and tribal or reservation lands, and those who identify as straight or heterosexual, who are not generally viewed as targets for PrEP campaigns and had lower levels of PrEP knowledge in this and other studies.[35,38,63,64] It will be critical to conduct sensitization activities in community settings as well as in health facilities to reach people who are not actively engaged in care or who anticipate stigma from healthcare providers. PrEP health education and outreach efforts should specifically focus on community health workers and other frontline public health workers, for their valuable connection to community and culture. Interventions designed by and for AI/AN communities, building on their culture, wisdom, and connectedness, are most likely to be effective [65–67].

Concerns about stigma within health facilities should also be addressed through evidence-based stigma reduction trainings for health facility staff, which use strategies such as information provision, skills-building activities, participatory learning, contact with stigmatized group, client empowerment, and structural or policy change [68]; building providers’ cultural competence and comfort taking sexual histories from all clients; and integrating PrEP into broader discussions of sexual health and wellbeing that address goals, rights, preferences, and concerns [69–72]. Messaging should emphasize that PrEP is for anyone who wants to feel protected from HIV, regardless of whether they perceive themselves to be “high risk”, as evidence suggests that PrEP reduces anxiety and HIV-related worry [57,73]. Clinical decision support tools integrated into medical records may help routinize sexual health discussions, HIV testing, and PrEP education [74–76]. Providers who are knowledgeable and nonjudgemental about PrEP may be able to help clients navigate challenges to access and affordability. Other service arrangements such as tele-PrEP services, pharmacy led PrEP services, and PrEP navigators may also improve access, affordability, and linkage to financial support [77–80]. Finally, education campaigns and provider counseling should help PrEP users address their sex partners’ misperceptions about PrEP and facilitate partner support for PrEP use upon disclosure. In sub-Saharan Africa, interventions focused on healthy communication skills and framing PrEP as protection for both partners in a couple have been shown to facilitate PrEP disclosure and support [81,82]. For AI/AN communities, models that build on cultural beliefs, including the emphasis on health and wellbeing, may be most successful.

## Conclusion

Findings from this study underscore the need for widespread sensitization about PrEP in AI/AN communities and healthcare settings. Although personal attitudes towards PrEP were relatively positive and levels of anticipated and perceived stigma were moderate, respondents expressed concerns about discussing PrEP with partners and health providers. Strategies to reduce stigma and build social support should reframe PrEP use as a part of overall sexual health and wellbeing, situate sexual health messaging on Indigenous pride and pre-colonial identities and roles, and build on patients’ connections with Indigenous culture and wisdom.

## Supporting Information

S1 File Supplemental Appendix 1: Study questionnaires. (PDF)

S1 Fig Participant responses to PrEP knowledge questions. (PDF)

S2 Fig Participant agreement to Stigmatizing PrEP Attitudes scale items. (PDF)

S3 Fig Participant agreement to Anticipated Stigma scale items. (PDF)

S4 Fig Participant agreement to Perceived Stigma scale items. (PDF)
