# Supplementary material for: PrEP knowledge, attitudes, and perceived barriers to access among American Indian/Alaska Native people in the US: Results from an online survey
Source: PLoS One. 2025 Apr 30;20(4):e0321422. doi: 10.1371/journal.pone.0321422 (PMC12043127; doi:10.1371/journal.pone.0321422)
Supplement: S1 File — (PDF) [file pone.0321422.s001.pdf]

Supplemental Appendix 1: Study Questionnaires

Screener survey

[DOB] What is your date of birth?

__ __ / __ __ / __ __ __ __ (MM/DD/YYYY)

[STATE] What state do you live in?
[Drop down box with U.S. States + option to select "I do not live in the U.S."]

[AIAN] Do you identify as an Indigenous, American Indian, Alaska Native, Native American, Native Hawaiian, or Pacific Islander person? (Select one)

1. Yes
2. No

[TRIBE] If yes, what Tribe are you a member of or a descendant from? [open ended]

[DISTRACTOR1] About how often do you visit social media sites, such as Instagram, TikTok, Snapchat, Facebook, or Twitter?

1. Every hour or more
2. More than once a day
3. About once a day
4. 3-5 days a week
5. 1-2 days a week
6. Every few weeks or less
7. I do not have a social media account

[ATTNCHK] The following question is designed to ensure that people are at a high attention level throughout the survey. Please select "Some days" as your answer to this question.

1. Every day
2. Some days
3. Rarely
4. Not at all

[ZIP] What is your zip code?
_ _ _ _ _ Enter your 5-digit zip code

[AGE] What is your age? ___(number)

[EMAIL]
Please enter your email address: ______________________ [OPEN TEXT]

The email address you provide is used only for the purposes of this survey and will **not** be sold or shared.

[EMAIL_VER]
Please verify your email address: ______________________

Main Survey

We encourage you to find a private place to complete the survey. Please do not complete the survey while driving.

Demographics. *Now we will ask questions about you and your background.*

1. What is your race/ethnicity? *(select all that apply*) **[REQUIRED]**
   - ☐ Indigenous
   - ☐ Native American
   - ☐ American Indian
   - ☐ Alaska Native
   - ☐ Native Hawaiian
   - ☐ Pacific Islander
   - ☐ White or Caucasian
   - ☐ Black or African American
   - ☐ Latinx
   - ☐ Hispanic
   - ☐ Asian
   - ☐ Not listed (Please specify:_)

Note: If Indigenous, Native American, American Indian, Alaska Native, Native Hawaiian, or Pacific Islander are not selected, this will be a flag for a potentially fraudulent response.

2. What is/are your gender identity/identities today? *(Select all that apply)*
   - ☐ Woman
   - ☐ Transwoman
   - ☐ Transman
   - ☐ Man
   - ☐ Nonbinary
   - ☐ Agender
   - ☐ Transgender
   - ☐ Genderqueer
   - ☐ Genderfluid
   - ☐ Two Spirit
   - ☐ Indigiqueer
   - ☐ Questioning or unsure
   - ☐ Other/not listed (please describe: _______)

3. How would you describe your sexual orientation? *(Select all that apply)*
   - ☐ Gay
   - ☐ Lesbian
   - ☐ Queer
   - ☐ Same-gender loving
   - ☐ Bisexual
   - ☐ Pansexual
   - ☐ Two Spirit
   - ☐ Indigiqueer
   - ☐ Asexual
   - ☐ Questioning
   - ☐ Straight (opposite-gender loving)
   - ☐ Other/Not listed (Please describe: ______)

4. What is (are) the gender(s) of your current and previous sexual partner(s)? *(Select all that apply)*
   - ☐ Woman
   - ☐ Transwoman
   - ☐ Transman
   - ☐ Man
   - ☐ Nonbinary
   - ☐ Agender
   - ☐ Transgender
   - ☐ Genderqueer
   - ☐ Genderfluid
   - ☐ Two Spirit
   - ☐ Indigiqueer
   - ☐ Questioning or unsure
   - ☐ Additional gender category/identity not listed (please specify: ____________________)

5. In what state do you live most of the time? (Select one) *[drop-down list of all states, including "Prefer not to answer" option]*

6. Which of the following best describes the place or area you live in most of the time? (Select one)
   - ☐ Urban
   - ☐ Suburban
   - ☐ Rural
   - ☐ Other (please describe:)

7. Which of the following best describes the place or area you live most of the time? (Select one)
   - ☐ Reservation/Tribal Lands
   - ☐ Not a Reservation/Tribal Lands

8. Where do you receive your healthcare most of the time? (Select one)
   - ☐ At an Indian Health Service facility
   - ☐ At a Tribal healthcare facility
   - ☐ At an Urban Indian healthcare facility
   - ☐ At a private healthcare facility
   - ☐ Other (Please specify: ________________________)

9. How strong is your connection to your indigenous culture or identity? (Select one)
   - ☐ Not strong at all
   - ☐ Somewhat strong
   - ☐ Neutral
   - ☐ Strong
   - ☐ Very strong

10. What are your three favorite Native foods? **[REQUIRED]**
    a. [open ended text]
    b. [open ended text]
    c. [open ended text]

11. What is the first Native medicine you were introduced to? [open ended text] **[REQUIRED]**

Section 3. HIV Pre-exposure Prophylaxis (PrEP) Knowledge. *Now we will ask you some questions to learn how much you already know about HIV Pre-exposure Prophylaxis, or PrEP.*

**Statement**	**Select one answer per statement:**
12. PrEP is a daily pill you can take to reduce your risk of becoming infected with HIV.	True/False/Don't know
13. You should not use PrEP if you don't know your HIV status.	True/False/Don't know
14. If you do not take PrEP consistently, there may not be enough medicine in your bloodstream to block the HIV virus.	True/False/Don't know
15. PrEP can be used to prevent STIs like gonorrhea, chlamydia, syphilis, herpes, and HPV.	True/False/Don't know
16. If you start taking PrEP, you will have to take it for the rest of your life.	True/False/Don't know
17. PrEP can be used as treatment by people who are living with HIV	True/False/Don't know
18. You must take an HIV test every 3 months while taking PrEP.	True/False/Don't know
19. There are many serious side effects of taking PrEP.	True/False/Don't know
20. Daily PrEP use can lower the risk of getting HIV from sex by more than 90%.	True/False/Don't know

Instructions: Before continuing to the next set of questions, please review the below information or watch this short video about HIV PrEP:
[Note: in the online survey, after watching the video or read the text below, respondent will be prevented from changing your answers to questions 13-21]

Video: **https://youtu.be/1_eo17YahCo**
Transcript:
PrEP, or pre-exposure prophylaxis, is a medicine that can prevent HIV.
PrEP is for people who are HIV negative and at risk of getting HIV. This includes people who have a sex partner with HIV, people who don't always use condoms, or people who have been diagnosed with an STD in the past 6 months.
PrEP is also for people who are HIV negative and sharing needles or other drug injection equipment, or have a drug-injecting partner with HIV.
PrEP is highly effective for preventing HIV through sex or injection drug use.
PrEP does NOT protect against other sexually transmitted diseases. Only condoms can protect against STDs such as syphilis or gonorrhea.
Visit your healthcare provider to see if PrEP is right for you. If you decide to take PrEP, you'll need to take it every day, or as prescribed by your healthcare provider.
For more information about PrEP, visit cdc.gov/hiv.

21. Are you currently or have you ever taken HIV Pre-exposure Prophylaxis (PrEP) for HIV prevention? *(Select one)*
    - ☐ Yes, I'm currently taking PrEP (*continue to Q22*)
    - ☐ Yes, I have taken PrEP previously but am not currently taking PrEP *(skip to Q25)*
    - ☐ No, I have never taken PrEP *(skip to Q27)*
    - ☐ Don't know *(skip to Q27)*
    - ☐ Prefer not to disclose *(skip to Q27)*

IF "Yes, I'm currently taking PrEP":

22. When did you start taking PrEP? (It is okay to estimate the date)
    ___ / _______[MM/YYYY]

23. How easy or hard is it to take PrEP?
    - ☐ Very easy
    - ☐ Easy
    - ☐ Neutral
    - ☐ Hard
    - ☐ Very hard

24. If hard or very hard: What makes it hard? [Open ended response]

After answering Q22-24, skip to Q30

IF "Yes, I have taken PrEP previously but am not currently taking PrEP":

25. About how many months did you take PrEP? (It is okay to estimate)
    ___ months

26. What are the reasons that you stopped taking PrEP? [Open ended response]

After answering Q25-26, skip to Q30

IF No/Don't know/Prefer not to disclose to #21:

27. Based on the information provided, how interested are you in using oral PrEP? Please choose the statement that is closest to how you feel. *(Select one)*
    - ☐ I do not think PrEP is a good option for me
    - ☐ I want to think more about PrEP before deciding if it is a good option for me.
    - ☐ PrEP may be a good option for me, but I am still unsure
    - ☐ I am ready to get screened for PrEP (i.e., get an HIV test and see if I am medically eligible)
    - ☐ I am ready to start taking PrEP.

28. If you wanted to get a prescription for PrEP, would you be able to? *(Select one)*
    - ☐ Yes
    - ☐ No
    - ☐ Not sure

29. If no or not sure to Q28, why wouldn't you be able to get a prescription? *(Select all that apply)*
    - ☐ I do not feel comfortable talking with my health care provider about PrEP
    - ☐ I do not live near a healthcare provider

- ☐ I do not have access to transportation to visit a health care provider
- ☐ I asked my provider or clinic for PrEP and was denied
- ☐ I feel that my health care provider would judge me if I asked about PrEP
- ☐ There isn’t privacy at my clinic, so people will know I’m on PrEP
- ☐ I do not know where to get PrEP
- ☐ I do not know if my clinic offers PrEP
- ☐ I cannot afford PrEP
- ☐ I cannot go to the clinic because of child or elder care
- ☐ I can’t leave work to go to the clinic when it is open
- ☐ Other (Please describe: ____________________________)

[go to next page]

Section 4. PrEP attitudes and beliefs.

Instructions: The following statements are about your attitudes towards and beliefs about PrEP. Please rate how strongly you agree with each statement. (Select one answer per statement)

Statement	Strongly Disagree	Disagree	Neither Agree nor Disagree	Agree	Strongly Agree
30. People should take PrEP					
31. People on PrEP are irresponsible					
32. Sex with someone on PrEP is risky					
33. PrEP users make smart decision to protect their health					
34. I wouldn't trust someone who told me they're on PrEP					
35. PrEP is used as excuse to have condomless sex					
36. PrEP users lie about taking it everyday					
37. People on PrEP are responsible					
38. People on PrEP sleep around					
39. People on PrEP can't control their sex drive					
40. I would feel ashamed to take PrEP pills in front of others.					
41. Someone taking PrEP should keep their pills hidden.					
42. I would have sex with someone who is taking PrEP.					
43. I would feel proud to take PrEP every day.					
44. People on PrEP are taking care of their health.					
45. My friends would be supportive of me taking PrEP.					
46. My family would be supportive of me taking PrEP.					
47. Someone taking PrEP would be treated unfairly by their doctors.					
48. People may experience problems when they tell their sex partner(s) they are taking PrEP.					

Instructions: This set of questions is about your community's attitudes toward and beliefs about PrEP. Please rate how strongly you agree with each statement. For the purposes of this survey, "community" includes the people and spaces where you live, work, play, and do ceremony. (Select one answer per statement)

Statement	Strongly Disagree	Disagree	Neither Agree nor Disagree	Agree	Strongly Agree
49. Most people in my community know what PrEP is.					
Note: *if Strongly Disagree/Disagree to above question, will have a prompt: "For the following questions, think about how your community would respond if they DID know what PrEP is."*					
50. Most people in my community think that people should take PrEP					
51. Most people in my community think that people on PrEP are irresponsible					
52. Most people in my community think that sex with someone on PrEP is risky					
53. Most people in my community think that PrEP users make smart decision to protect their health					
54. Most people in my community wouldn't trust someone who told them they're on PrEP					
55. Most people in my community think that PrEP is used as excuse to have condomless sex					
56. Most people in my community think that PrEP users lie about taking it everyday					
57. Most people in my community think that people on PrEP are responsible					
58. Most people in my community think that people on PrEP can't control sex drive					
59. Most people in my community would feel ashamed to take PrEP pills in front of others.					

Statement	Strongly Disagree	Disagree	Neither Agree nor Disagree	Agree	Strongly Agree
60. Most people in my community think that someone taking PrEP should keep their pills hidden.					
61. Most people in my community would have sex with someone who is taking PrEP.					
62. Most people in my community would feel proud to take PrEP every day.					
63. Most people in my community think that people on PrEP are taking care of their health.					
64. Most people in my community would assume that someone taking PrEP is sleeping with many people.					
65. Most people in my community would assume I was HIV positive if I took PrEP					
66. Most people in my community would assume I was Two Spirit, lesbian, gay, transgender, queer/questioning, or other non-cisgender/non-heterosexual identity (LGBTQ2S+) if I took PrEP					
67. Most people in my community would assume I use drugs if I took PrEP					

[go to next page]

**Section 5. Experience with Health Care.** *This section is about your experience with doctors and other health care providers where you seek health care* *most of the time. Please rate how strongly you agree with each statement. (Select one answer per statement)*					
**Statement**	**Strongly Disagree**	**Disagree**	**Neither Agree nor Disagree**	**Agree**	**Strongly Agree**
68. Where I seek care, doctors and health care workers sometimes hide information from patients who belong to my ethnic group					
69. Where I seek care, doctors have the best interests of people of my ethnic group in mind					
70. Where I seek care, people of my ethnic group should not confide in doctors and health care workers because it will be used against them					
71. Where I seek care, people of my ethnic group should be suspicious of information from doctors and health care workers					
72. Where I seek care, people of my ethnic group cannot trust doctors and health care workers					
73. Where I seek care, doctors and health care workers treat people of my ethnic group like "guinea pigs."					
74. Where I seek care, people of my ethnic group receive the same medical care from doctors and health care workers as people from other groups					
75. Where I seek care, doctors and health care workers do not take the medical complaints of people of my ethnic group seriously					
76. Where I seek care, people of my ethnic group are treated the same as people of other groups by doctors and health care workers					
77. Where I seek care, I have personally been treated poorly or unfairly by doctors or healthcare workers because of my ethnicity.					

Section 6. Community Attitudes and Beliefs about HIV

Please rate how strongly you agree with each statement. For the purposes of this survey, "community" includes the people and spaces where you live, work, play, and do ceremony. (Select one answer per statement)

Statement	Strongly disagree	Disagree	Neither Agree nor Disagree	Agree	Strongly Agree
78. Most people in my community treat people with HIV like outcasts					
79. Most people in my community believe a person who has HIV is dirty					
80. Most people in my community think a person with HIV is disgusting					
81. In my community, most people with HIV are rejected when others find out					
82. Most people in my community are uncomfortable around someone with HIV					

Section 7. Community Attitudes and Beliefs about Gender and Sexual Orientation

This section is about your community's attitudes and beliefs about gender and sexual orientation. Please rate how strongly you agree with each statement. For the purposes of this survey, "community" includes the people and spaces where you live, work, play, and do ceremony. (Select one answer per statement.)

Question	Strongly disagree	Disagree	Neither Agree nor Disagree	Agree	Strongly agree
83. Most people in my community think that no one should experience job discrimination because of their sexual orientation					
84. Most people in my community think that all people should be able to have any kind of consensual sex in private without being fined or arrested					
85. Most people in my community think that gender and sexual minorities should be allowed to express their opinions in public					

86. Most people in my community think that gender and sexual minorities should be allowed to be school teachers					
87. Most people in my community think that same-sex couples should be able to attend workplace social events together as partners					
88. Most people in my community think that people are either men or women					
89. Most people in my community think that a man should be able to dress like a woman, if he chooses					
90. Most people in my community think that a woman should be able to present herself as a man in public, if she chooses					

Section 8. Community Attitudes and Beliefs about People Who Use Drugs. *This section is about your community's attitudes towards and beliefs about people who use drugs. Please rate how strongly you agree with each statement. For the purposes of this survey, "community" includes the people and spaces where you live, work, play, and do ceremony. (Select one answer per statement.)*

Statement	Strongly Disagree	Disagree	Neither Agree nor Disagree	Agree	Strongly Agree
91. Most people believe that people who use drugs cannot be trusted					
92. Most people believe that people who use drugs are dangerous					
93. Most people would not accept a person who uses drugs as a close friend					
94. Most people would feel that drug use is a sign of personal failure					
95. Most people will take a person less seriously if they are known to use drugs.					
96. Most people think less of a person that uses drugs					
97. Most people would treat a person who uses drugs just as they would treat anyone else					
98. Most employers will not hire a person who uses drugs					

Section 9. Experiences of Discrimination. *The next set of questions is about your experiences of discrimination.*

In your day-to-day life, how often do the following things happen to you? *(Select one answer per statement)*

	**Almost every day**	**At least once a week**	**A few times a month**	**A few times a year**	**Less than once a year**	**Never**
99. You are treated with less courtesy than other people are						
100. You are treated with less respect than other people are						
101. You receive poorer service than other people at restaurants or stores						
102. People act as if they think you are not smart						
103. People act as if they are afraid of you						
104. People act as if they think you are dishonest						
105. People act as if they're better than you are						
106. You are called names or insulted						
107. You are threatened or harassed						

If "a few times a year" or more frequently to at least one of the above questions,

108. What do you think is the main reason for these experiences? (*Select all that apply*)

- ☐ Your Indigeneity
- ☐ Your Ancestry or National Origins
- ☐ Your Gender
- ☐ Your Race
- ☐ Your Age
- ☐ Your Religion
- ☐ Your Height
- ☐ Your Weight
- ☐ Some other Aspect of your Physical Appearance
- ☐ Your Sexual Orientation
- ☐ Your Education or Income Level
- ☐ Your HIV status
- ☐ Your PrEP use
- ☐ Others thinking that you use illicit drugs or prescription medication illicitly
- ☐ A physical disability
- ☐ Your shade of skin color
- ☐ Other (specify:)

Thank you for taking this survey. All surveys will be analyzed for validity and those deemed valid will receive their incentive within 2 weeks.
