# Supplementary figures and images for: PrEP knowledge, attitudes, and perceived barriers to access among American Indian/Alaska Native people in the US: Results from an online survey
Source: PLoS One. 2025 Apr 30;20(4):e0321422. doi: 10.1371/journal.pone.0321422 (PMC12043127; doi:10.1371/journal.pone.0321422)

**Supplemental Figure 1:** Participant responses to PrEP knowledge questions

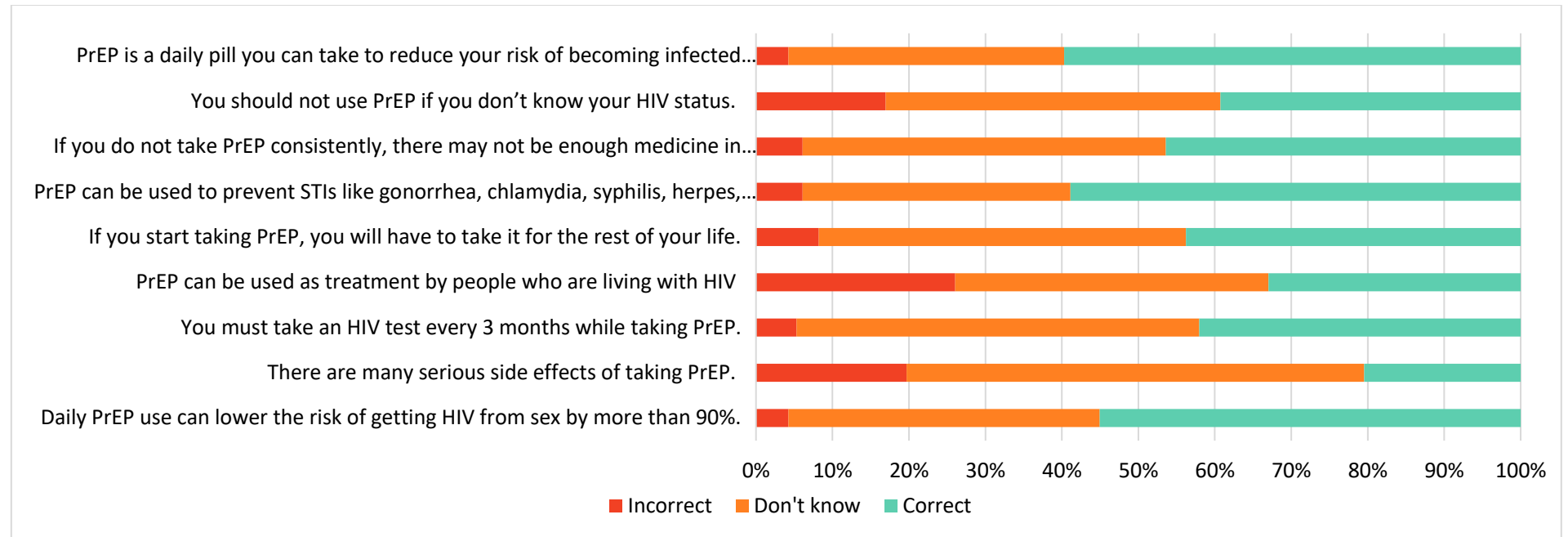

Supplement: S1 Fig — (PDF) [file pone.0321422.s002.pdf]

**Supplemental Figure 2:** Participant agreement to Stigmatizing PrEP Attitudes scale items.

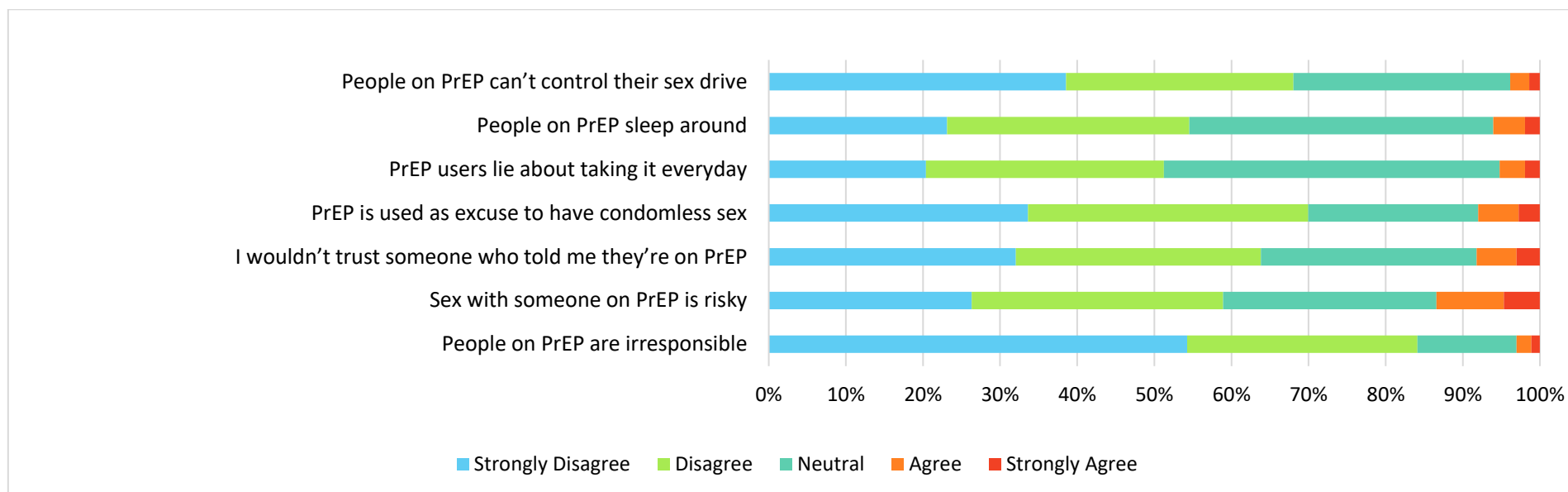

Supplement: S2 Fig — (PDF) [file pone.0321422.s003.pdf]
