# Supplementary material for: PrEP knowledge, attitudes, and perceived barriers to access among American Indian/Alaska Native people in the US: Results from an online survey
Source: PLoS One. 2025 Apr 30;20(4):e0321422. doi: 10.1371/journal.pone.0321422 (PMC12043127; doi:10.1371/journal.pone.0321422)

Supplemental Figure 3: Participant agreement to Anticipated Stigma scale items. *For items labeled with an asterisk (*), responses have been reverse-coded so that the right side of the distribution, coded in red indicates more stigmatizing responses, and the left side of the distribution, coded in blue, indicates less stigmatizing responses.*

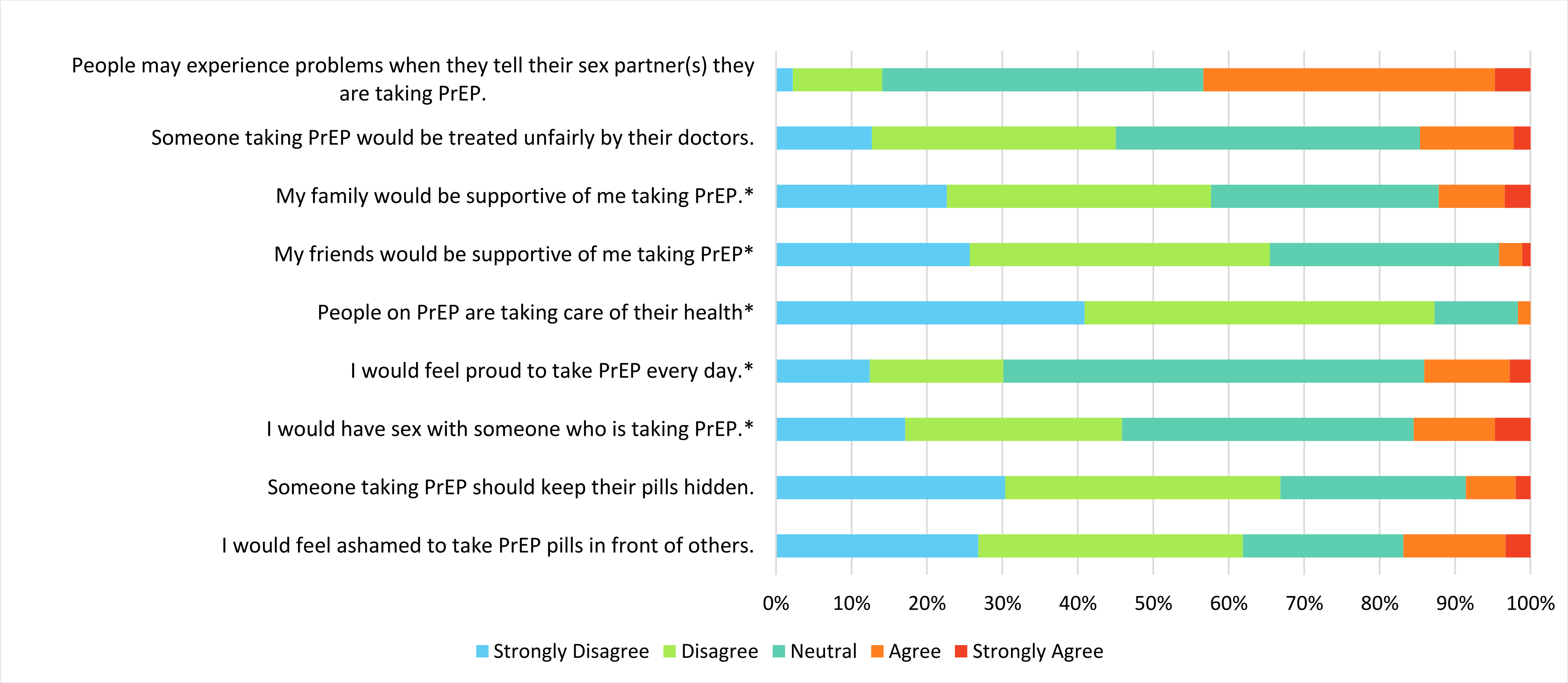

Supplement: S3 Fig — (PDF) [file pone.0321422.s004.pdf]
